# Supplementary material for: A guide to selecting high-performing antibodies for TMEM175 (UniProt ID: Q9BSA9) for use in western blot, immunoprecipitation, and immunofluorescence
Source: F1000Res. 2026 Jun 16;15:942. [Version 1] doi: 10.12688/f1000research.182354.1 (PMC13560852; doi:10.12688/f1000research.182354.1)
Supplement: Peer review history

# Reviewer report: Reviewer response for version 1

**DOI:** 10.5256/f1000research.201286.r506574

**Published:** 2026-08-20

**Authors:** Christian Tiede

**Affiliations:** 1 University of Leeds, Leeds, England, UK

This study compares six commercially available TMEM175 antibodies for use in Western blotting, immunoprecipitation, and immunofluorescence assays using HeLa wild-type and knockout cells. In addition, the cell lines A172, DMS-53, U-87 MG, and SH-SY5Y were analyzed by Western blotting, as their TMEM175 RNA expression levels exceeded the author’s cut-off of 2.5 log _2_. The study highlights substantial differences among the antibodies across all three applications and provides valuable guidance for selecting the most suitable reagent for specific experimental needs. Figure 3 is particularly useful for informing antibody selection. The article is suitable for approval.

 However, the reporting of secondary antibodies could be improved. Information such as catalog numbers should be included in the figure legends, the Methods section, or a dedicated antibody summary table. Presenting these details only in the "Cell Lines and Antibodies" section makes them unnecessarily difficult to locate. In addition, the use of Veriblot (Abcam, cat. no. ab131366) for immunoprecipitation experiments is recommended, as it does not detect denatured IgG heavy and light chains. This approach has been successfully adopted in similar antibody characterization studies and enables clearer interpretation of immunoprecipitation results.

Are sufficient details of methods and materials provided to allow replication by others?

Yes

Is the rationale for creating the dataset(s) clearly described?

Yes

Are the datasets clearly presented in a useable and accessible format?

Yes

Are the protocols appropriate and is the work technically sound?

Yes

Reviewer Expertise:

Phage display, western blotting, immunoprecipitation

I confirm that I have read this submission and believe that I have an appropriate level of expertise to confirm that it is of an acceptable scientific standard.

# Reviewer report: Reviewer response for version 1

**DOI:** 10.5256/f1000research.201286.r495098

**Published:** 2026-07-17

**Authors:** Sian E. Evans

**Affiliations:** 1 University of Leicester, Leicester, England, UK

The authors describe the characterization of six commercial antibodies against TMEM175 for use in western blotting, immunoprecipitation, and immunofluorescence, by comparing experimental readouts obtained from wild-type and knockout cell lines.

 To identify a suitable cell line expressing sufficient levels of the target protein, the authors used the DepMap transcriptomics database to screen for cell lines with TMEM175 expression levels greater than 2.5 log₂ transcripts per million +1 (TPM +1). HeLa cells were selected for antibody characterisation, as they express TMEM175 at 3.8 log₂ TPM +1. A TMEM175 knockout HeLa cell line was obtained from Abcam.

 The manuscript clearly explains that not all antibodies are recommended for all three applications. The methods are described in sufficient detail, and the inclusion of links to openly accessible materials and step-by-step protocols on Protocols.io will support reproducibility. The authors also appropriately acknowledge the limitations of their characterisation platform.

 Table 3 is a particularly valuable addition to the Data Note, especially for researchers who are less familiar with antibody-based techniques. It provides clear guidance on the expected outcomes for each application. One minor suggestion would be to revise the wording in the immunofluorescence (IF) section of Table 3: replacing “Target protein not detected” with “Non-specific protein binding” may provide a more accurate description of the observed result.

Are sufficient details of methods and materials provided to allow replication by others?

Yes

Is the rationale for creating the dataset(s) clearly described?

Yes

Are the datasets clearly presented in a useable and accessible format?

Yes

Are the protocols appropriate and is the work technically sound?

Yes

Reviewer Expertise:

multiplexed immunofluorescence, western blotting, patient-derived explants, cancer

I confirm that I have read this submission and believe that I have an appropriate level of expertise to confirm that it is of an acceptable scientific standard.
